# Reviewing the evidence base for social recovery in mental health service provision: A protocol for a scoping review

**DOI:** 10.1371/journal.pone.0324249

**Published:** 2025-05-21

**Authors:** Calvin Swords, Michael John Norton, Alan Maddock

**Affiliations:** 1 School of Applied Social Studies, University College Cork, Cork, Ireland; 2 Adult Continuing Education, University College Cork, Cork, Ireland; 3 Department of Health Psychology, School of Population Health, Royal College of Surgeons in Ireland, Dublin, Ireland; National Institutes of Health, University of the Philippines Manila / De La Salle University, PHILIPPINES

## Abstract

**Introduction:**

The concept of recovery within mental health service delivery is no longer a new concept across the westernised world. However, its development in terms of implementation policy and practice has remained challenging for all stakeholders. This has focused on personal recovery being unattainable for many individuals due to neoliberalism and individualism. Consequently, one argument which is beginning to build is the need to focus on the idea of social recovery, a relatively new concept. However, no synthesis of social recovery’s evidence base in relation to mental health service provision has taken place. This protocol provides a detailed plan of how a scoping review would be undertaken and completed to examine this evidence.

**Methods and analysis:**

Adopting Arksey and O’Malley’s framework, A Preferred Reporting Items for Systematic Reviews and Meta-Analysis compliant scoping review has been chosen. This includes a five-stage approach to completing scoping reviews. This includes the search terms that will be used. It also details the variety of databases (CINAHL, EBSCO, Jstor, OVID SP, PsychINFO, PubMed, RCNi, Science Direct, Web of Science and Scopus) and other sources including repositories (Cochrane Online Library, ETHos, nz.research.org.nz, ProQuest, National ETD Portal, Google, Google Scholar and ResearchGate). Inclusion and exclusion criteria are illustrated in this protocol. Given that the concept of social recovery is relatively new, no search range was chosen.

## Introduction

Until the 1980s, recovering from mental distress was informed by a medical evidence base which focused on identifying symptoms, reducing them through medication, and returning to a person’s functioning baseline [1–3]. A key contributor to the expansion beyond this idea dates to a study in 1967 by the World Health Organisation (WHO) [4]. Focusing on schizophrenia, the longitudinal findings highlighted a range of variations in outcomes for those living with mental illness. In other words, the focus of recovery was on the reduction of clinical symptoms and deficits which were impacting on the daily functioning of individuals. More specifically, only 25–65% of participants experienced partial to full recovery [4]. Consequently, it was one of the first studies to highlight the need to focus beyond medication when viewing an individual’s recovery from mental illness. It was also one of the first indications that the biomedical paradigms efficacy was falling short of the desired outcomes in mental health.

A significant response to such findings was seen in the rise of the social movement of disaffected users of services in the 1980s [5]. In 1988, Deegan [6] provided an insight into her own recovery process, claiming that recovery could be built through constructing a new identity and sense of self [7,8]. Individuals such as Deegan believed that recovery should be about overcoming the outcomes associated with being identified as a ‘psychiatric patient’ [1,7,8]. In other words, recovery should be about constructing an identity which was beyond their mental health diagnoses and the impact of this label on everyday life. Consequently, the concept of personal recovery was introduced to mental health discourse:

*“It is a way of living a satisfying, hopeful and contributing life, even with limitations caused by illness”*

[9, p15]

Recovery has been conceptualised as an individualised journey taken by someone who is experiencing mental health challenges. It is subjective and unique, experienced by the individual [5,7,10]. In response to the biomedical perspective, personal recovery provided a broader and more inclusive conceptualisation of what recovery should be for those using and providing services [3]. It provided the potential for hope and empowerment for those receiving services that they could live an independent and fulfilling life regardless of their diagnosis [1,5,7]. This interpretation of recovery became central to mental health services in the westernised world [3,5,11].

In terms of reflecting on the developments associated with recovery in mental health, there are multiple competing perspectives [7,10,12]. Recovery is possible with or without a biomedical focus. It can involve becoming an employee, commencing an educational course, or being able to live independently. However, the key concern is whether recovery-orientated services have implemented the ethos of personal recovery into practice. Change is still necessary [3,12–14].

### The evidence base for personal recovery

In response to these developments associated with conceptualising recovery, there has been a focus on developing the knowledge and evidence-base surrounding personal recovery [7,14,15]. A systematic review, combined with a modified narrative synthesis was undertaken in 2011 sought to identify the key themes necessary for promoting positive outcomes for personal recovery [16]. This led to the creation of the CHIME framework, **C**onnection, **H**ope, **I**dentity, **M**eaningful Role and **E**mpowerment [16]. There were 1100 papers considered and reviewed, with these five themes being central to a positive outcome for each individual on their recovery journey.

Although there have been important developments regarding building the evidence base for personal recovery, its translation into practice has remained inconsistent [3,12,14,17–19]. One argument has been the lack of understanding of the recovery principles associated with a recovery-orientated approach [14]. A further reason claimed in the literature [13] is the lack of resources and capacity in services to support partnerships between service users, families/carers, and service providers. A reoccurring argument seen in the literature for these barriers is the biomedical model’s dominance within service culture [3,12,19,20].

### Neoliberalism and recovery

The need for governments to ensure cost effectiveness has also contributed to the reality of recovery-orientated services [13]. The focus on the economic case for personal recovery supports a core argument within the literature regarding the impact of neoliberalism [21–23]. In the late twentieth century there has been a shift towards economic and political focus in the western world on “deregulation, efficiency and profit-making” [24, p277]. Focusing on the philosophy of neoliberalism, it claims that market capitalism can lead to people being liberated from state control through *“devolved power, individual responsibility and choice”* [21, p75].

A paradox has developed in relation to personal recovery [21,25]. This is seen in the promised individual freedoms attached to personal recovery being constrained in a system which offers unattainable ideas in relation to *“free and rationale choice”* [21, p76]. Essentially, neoliberal agendas have contributed to recovery shifting towards individual responsibility and choice [21,24–27]. Subsequently, once an individual enters society, they are faced with many barriers to achieving fulfilment in their lives. These include housing, employment and poverty [13,28,29].

### Social and personal recovery

Social recovery has not received the same level of coverage or attention as personal recovery [11,13,30]. One reason for this is the overlap and misinterpreted understanding of social recovery when discussed alongside personal recovery [13,31]. There is also growing realisation that there is a need for more of a focus and understanding of the social factors which contribute to a positive recovery journey [11,13,32]. In 2006, a paper explored the need for an understanding of “recovery in a social context” with a recognition that interpersonal and social processes are key considerations when mapping an individual’s recovery journey [33, p63].

In 2018, the first paper was published which argued for the place of social recovery when seeking to achieve positive outcomes in relation to personal recovery [13]. Social recovery focuses on the collective culture within our society, identifying an individual’s opportunity for connectedness and social capital. Social capital includes recovery capital. In terms of outcomes in relation to social recovery, an individual being an active and participating member of society, otherwise known as active citizenship, with a sense of belonging, are core tenets of this perspective [11,18].

Some parallels between social and personal recovery have led to the former being often overlooked [11,13,18]. Social recovery is focused on the interactions people experience with their social world on their personal recovery journey. Subsequently, it also takes into consideration the interdependence of the relationships built within these everyday interactions [11,13]. It is in these relationships that opportunities for connectedness, social, and recovery capital can take place. These openings are influenced and determined by the structural and cultural factors of the socio-economic politics of each country or region [13].

In 2020, Norton and Swords [11] undertook a critical literature review reflecting on the first accounts of personal recovery [6,9]. Interestingly, the findings from Norton & Swords [11] identified that service users had shifted their focus from personal to social recovery in terms of their views of what recovery was. In other words, they spoke of the need for the necessary social factors to be developed and available for fulfilment to be achieved in their lives [11].

Norton and Swords’ [11] critical literature review built on the paper from Ramon [13]. The review posed the question, “what are service users’ views on the recovery concept within mental health services?”. The focus of the review was to see if there had been a shift in service user’s views of recovery since Deegan [6] and Anthony [9] in the late twentieth century. A systematic, six-step approach was taken to the critical literature review [34]. This approach is a strategy which considers some important aspects of systematic approaches, but also includes elements of more traditional narrative reviews [34]. The six-steps included constructing a clear description of what the research topic was seeking to explain. This involved a series of questions being asked of the researcher regarding ‘what’ and ‘why’ rationales for undertaking the research [34].

The findings from Norton and Swords [11] included four articles [35–38]. Through the iterative process of thematic analysis [39,40], there were six core influencers inductively constructed - health, economics, social connection/interaction, housing, personal relationships, and support [11]. These influencers were found to be the key influencers of service users’ [35–38] perspectives on the process of social recovery. Table 1 provides an explanation of each of these influencers:

**Table 1:** An overview of Norton & Swords [11] six key influencers of social recovery.

Key Influencers	Explanation
Health	Optimal health includes physical, social, and mental health. This contributes to overall well-being.
Economics	Economic stability in the context of social recovery focuses on the steps required for this stability – includes the role of employment and education.
Social Connection/Interaction	Opportunities for social interaction can lead to connections and friendships.
Housing	Appropriate accommodation can lead to experiences of stability, independence, and safety.
Personal Relationships	Repairing, sustaining, and improving the personal relationships is central to the recovery journey process.
Support	Social supports include voluntary supports within an individual’s community. Purchased supports refer to formal supports such as peer support workers.

Norton and Swords [11] provided a further step towards understanding the philosophy of social recovery. The six influencers identified in the final set of articles [35–38] provides core pillars for further exploration. In conjunction with the six influencers [11], the literature that has been briefly discussed so far has identified several additional potential influencers associated with social recovery. These are listed as follows, including references to the associated studies:

- Hope [16]
- Empowerment [16]
- Independence [1,5,7]
- Fulfilment/Social Wellbeing [1,5,7]
- Life Skills [1,5,7]
- Quality of Life [1,5,7]
- Connectedness [16]
- Social Integration [13]
- Active Citizenship [13]

There is a need for the evidence base in relation to social recovery to be explored in more detail, especially regarding the breadth of the literature and evidence base. Furthermore, reflecting on the study from Norton and Swords [11], the focus was on exploring the views of service users in relation to the concept of recovery as a personalised journey. It was found that people’s experiences illustrated a need for a focus on social recovery. Consequently, this protocol and scoping review will explore what exactly is the evidence base for social recovery.

### Rationale for study protocol

This protocol has been devised due to the dearth of evidence which focuses on the evidence-base for social recovery in mental health. In terms of personal recovery, there have been several reasons for the shortcomings of the approach, which were discussed in the previous section [13,21,25]. Furthermore, Norton and Swords [11] highlighted that service users’ perspectives of recovery had shifted from a focus on personal to social recovery [35–38].

For service users, there recovery journey has been hindered by the lack of focus and understanding of social recovery. Essentially, there is a need to focus on exploring whether recovery-based approaches have considered the influencers identified by Norton and Swords [11] and the other above listed possible influencers. Therefore, the purpose of this review protocol is to develop a clear understanding of social recovery, its principles, and evidence-base.

### Aims & objectives of the proposed scoping review

This article will illustrate the protocol for a scoping review which seeks to explore and understand the evidence base for social recovery in mental health. The objectives of this review will include the following:

1. To identify and outline specifically what the core concepts underpinning social recovery are based on the research evidence available.
2. To collate and analyse the definitions, approaches, types, and models of social recovery evident in the literature.
3. To scope the evidence base within the literature available in relation to the effectiveness of recovery approaches in supporting social recovery.
4. To explore the evidence base for the six influencers identified by Norton and Swords [11] in relation to social recovery.

It is important to note that our aim is identify and outline what is in the literature. We do recognise as an authorship that our objectives are broad but believe this is necessary due to the nascent nature of the evidence base. During the scoping review process, if the broad nature of the study does impact on our aims and objectives, we will acknowledge this in the findings.

## Methods & analysis

The scoping review undertaken will support the identification of the breadth of evidence available in past and current literature in relation to the concept of social recovery. The review will be informed and devised based on the Preferred Reporting Items for Systematic Reviews and Meta-Analysis for Scoping Reviews (PRISMA-ScR). PRISMA-ScR is the standard reporting guidelines which were specifically devised for scoping reviews [41]. The scoping review will be methodologically underpinned by Arksey and O’Malley’s framework, which includes five phases: 1) Identifying the research question, 2) Identifying relevant studies, 3) study selection, 4) charting of the data and finally 5) collating, summarising, and reporting of the results [41]. This protocol has been registered with OSF registries on 18 September 2022 and can be freely accessed from their website. The authors commenced this project in July 2023.

### Stage 1: Identifying the research question

Scoping reviews seek to explore the breadth of a concept. Consequently, the research questions should be broad and exploratory [41]. Scoping reviews are different to systematic reviews which focus on depth, rather than breadth. Given the limited evidence already published regarding social recovery, a scoping review was more appropriate than a systematic review. The researchers will seek to answer the following question, what does the evidence base tell us about the concept of social recovery in relation to mental health?

### Stage 2: Identifying relevant studies

Both published and grey literature will be explored through the relevant databases related to mental health. The following databases will be included: CINAHL, EBSCO, Jstor, OVID SP, PsychINFO, PubMed, RCNi, Science Direct, Web of Science and Scopus. In conjunction with this, other sources including repositories will be included for identifying relevant studies: Cochrane Online Library, ETHos, nz.research.org.nz, ProQuest, National ETD Portal, Google, Google Scholar and ResearchGate. To guarantee all studies relevant to the proposed research are included, a reference search will be undertaken on included studies.

### Stage 3: Study selection

The PICO method (Table 2) will be used to construct search strings which will support the research team in exploring the breadth of relevant studies to answer the research question posed [42]. The PICO method constitutes of 4 criterions for building a search statement – **P**atient or **P**opulation, **I**ntervention or **I**ssue, **C**omparison and **O**utcome or Measurement. Table 2 provides a breakdown of the research question using the PICO method.

**Table 2:** PICO used for constructing search statements.

Population	Service Users, Family Members/Carers, Service Providers.
Intervention	Social Recovery – focus on six influencers from [11]: ■ Health ■ Economics ■ Social connection/interaction ■ Housing ■ Personal relationships ■ Social & Purchased Support Also, other social recovery related outcomes noted from literature discussed: ■ Hope ■ Empowerment ■ Independence ■ Fulfilment/Social Wellbeing ■ Life Skills ■ Quality of Life ■ Connectedness ■ Social Integration ■ Active Citizenship
Comparison	Other forms of Recovery
Outcome	Social integration or inclusion in life in terms of personal recovery

Therefore, in the selected databases and repositories examined, the search will comprise of the following search terms presented in Table 3 below:

**Table 3:** Use PICO to break down your research question.

Concept 1	Health and Social Care Professionals, Policymakers, Service Users, Service Providers, Family members, Carers.
Concept 2	Social Recovery (Interchange with the listed outcomes/influencers)
Concept 3	Social Inclusion
Concept 4	Mental Health Services

Table 4 above provides an overview of the different search terms that will be used. The authors will use relevant Boolean operators in the databases including ‘AND’ in order to achieve the desired results for the proposed scoping review. Furthermore, there will be no time limits set on the scoping review in terms of the search range given that the idea of social recovery is relatively new to the area of mental health. However, there will be no literature included beyond 31 July2025. In the first phase of screening the literature (title of papers), the breakdown of the research question will be devised by using the PICO framework. Subsequently, in instances where the search will need further screening to determine a studies relevance to the research question, criterion will be applied from the inclusion/exclusion table created (Table 5):

**Table 4:** Identify search terms, synonyms, variant spellings.

Concept 1 (search terms)	Concept 2 (search terms)	Concept 3 (search terms)	Concept 4 (search terms)
1) Health & Social Care Professionals Or Health Professionals Or Social Care Professionals Or Medical Personnel Or Health Practitioners Or Health Personnel Or Health Specialist	Social Recovery Or Recovery and Social Support Or Social Inclusion and Recovery Or Health Or Economics Or Social Connections/Interactions Or Housing Or Personal Relationships Or Purchased Support Or Hope Or Empowerment Or Independence Or Fulfilment Or Social Wellbeing Or Life Skills Or Quality of Life Or Connectedness Or Social Integration Or Active Citizenship Or Citizenship	Social Inclusion Or Mental Health Social Inclusion Or Citizenship	Mental Health Services Or Mental Health Context Or Mental Health institutions Or Mental Asylums
2) Policymakers Or Policy Analyst Or Government*	Same as above	Same as above	Same as above
3) Service User Or Client Or Patient Or Lived Experience Consumer Or Participant*	Same as above	Same as above	Same as above
4)Family Or Family Perspective Or Family Involvement Or Carer Involvement Or Carer Perspective	Same as above	Same as above	Same as above

**Table 5:** Inclusion/exclusion criteria.

Inclusion	Exclusion
Quantitative/Qualitative/Mixed Method Peer Reviewed Studies, Reports, Dissertations, Literature Reviews [of any kind], Discussion Papers, Perspective Papers	Editorials and Periodicals
English Language	Papers not written in English
Papers which mentioned social recovery or one of its influencers in the title/abstract	Titles and abstracts which do not include the word social recovery
Focus of paper is on social recovery in relation to mental health service delivery, provision, policy, and development. So must consider one or more of the six influencers: ■ Health ■ Economics ■ Social connection/interaction ■ Housing ■ Personal relationships ■ Social & Purchased Support [11] Also, other social recovery related outcomes noted from literature discussed: ■ Hope ■ Empowerment ■ Independence ■ Fulfilment/Social Wellbeing ■ Life Skills ■ Quality of Life ■ Connectedness ■ Social Integration ■ Active Citizenship	Papers which focus on Personal Recovery but do not refer to one of the six influencers outlined by [11]
Mental Health	Other Areas of Health

The inclusion/exclusion criteria are specific without being too exhaustive. The rationale for this decision is the concern that the evidence base is very limited. For the value and purpose of this paper, a consideration of papers beyond mental health may be necessary when constructing the findings and implications of the paper.

In terms of the reporting procedure of the scoping review proposed, PRISMA’s extension for scoping reviews (PRISMA-ScR) will be used as a checklist [S1 Checklist]. Subsequently, the authors will use a flow diagram for the benefit of others to replicate this protocol.

In terms of the timeline for the retrieval of papers, it is expected to commence on 1 August 2025. This process will be concluded through a narrowing down process using the above outlined search strategy by the 30 November 2025.

### Stage 4: Charting the data

The purpose of a scoping review is to illustrate the breadth of evidence in relation to a specific research question posed. An important part of any scoping review is how the authors will visually present the findings. The studies included in the review will be displayed in a table format, using the following headings:

- Authors
- Year of Publication
- Country where study was conducted or affiliation of the first author
- Journal
- Targeted Audience
- Format of Paper – Dissertation, empirical or report.
- Setting – acute, community, residential.
- Aim of the study
- Study Design
- Methodology
- Theoretical Framework
- Data Collection Methods
- Sample & Sample Size
- How Social Recovery is defined
- Evidence of Social Recovery Outcomes
- Strengths and Limitations regarding Social Recovery
- Recommendations

The authors will construct a visual graph from connectedpapers.com to support the process of displaying the breath of the literature regarding the evidence base for social recovery. This will include identifying lead papers which will be found through the process of selecting the most referenced paper from each study’s reference list.

### Stage 5: Collating, summarising and reporting results

A narrative report, underpinned by thematic analysis approaches will be used to report the results of this proposed scoping review [39,40]. In line with this type of review, no data synthesis will take place. Consequently, the focus will be on breadth of the evidence base for or against social recovery, rather than focus on issues of rigor, validity, and study quality [43]. The authors will all participate in the collating, summarising, and reporting of the results. In terms of the searching process, the authors will first search each database individually, then there will be a joint search undertaken to cross-examine the results. If there are any disagreements between authors regarding the results, PRISMA’s extension for scoping reviews (PRISMA-ScR) will be used to refocus the group and overcome such issues if they present during the process. Finally, the reviewers will seek to explore and capture the gaps evident within the literature relating to the concept of social recovery. The authors estimate that the review will be completed by January 2026.

## Ethics & dissemination

This study is a scoping review. Consequently, it is not necessary to have ethical approval. The protocol will be stored on OSF Registries, where it will be freely accessible to anyone interested in this research. The results of the protocol will be used in peer-reviewed publications.

## Patient & public involvement

For this scoping review, there was no involvement from patients and/or public in the review process. However, one of the authors does have lived experience of services, which they will utilise when undertaking and completing the scoping review process. Furthermore, the authorship plan to share the initial findings with key perspectives within the lived experience field of research and practice. This will support the authorship in making sure the results of the review is informed and shaped by lived experience involvement beyond the authorship.

## Discussion

Mental health recovery is a concept with many competing agendas in relation to how it should be understood, researched, and applied in practice [5,10,37]. Personal recovery’s ethos and value has been eroded away by a neoliberal political agenda seen across the westernised world [21–23]. The responsibility of recovery is viewed as that of those receiving services for their mental health [25]. The continued focus on the individual, rather than the social structures influencing agency (government policy) to provide the appropriate conditions for recovery has remained a subordinated debate in current mental health discourse.

This scoping review seeks to explore the evidence-base of social recovery. This position on recovery seeks to focus beyond the individual, considering the social circumstances and processes necessary for successful recovery [13]. By identifying and exploring the evidence base, this can help inform all stakeholders on how social recovery could be implemented and integrated in service delivery. This has the potential to contribute to a new episteme regarding the concept of recovery.

This is possibly a strength of the overall review in terms of highlighting a gap in the mental health literature regarding recovery. Also, there are three authors of this review, resulting in an increased level of rigor in terms of devising, collating, analysing, and reporting the breath of evidence for social recovery. In terms of the limitations, all three authors have a pro-social recovery orientation due to their professional backgrounds, two are social workers, and the other, a peer support worker. To mitigate against this, the authors have and will take this into consideration when completing each stage of the scoping review. Another potential limitation of the study methodology is that inclusion and exclusion criteria may lead to relevant studies being omitted if for example, they do not include ‘social recovery’ in their abstract or title. However, the authors will review the reference lists of papers to mitigate against this risk to this scoping review. Finally, given that social recovery is still a relatively new concept, the conclusions drawn from the review may not provide generalisations for the authors to make regarding the evidence-base for social recovery.

### Strengths and limitations of proposed review

- This is a scoping review, therefore no quality assessment of the studies selected will be undertaken.
- The scoping review planned will be comprehensive, including peer-reviewed and grey literature.
- There are three researchers involved in this review. It is hoped that this will enhance the quality of the review.
- There was no patient and public involvement in creating this protocol, which may weaken the quality of this paper and subsequent scoping review.

## Supporting information

S1 Checklist S1 PRISMA-P-checklist. (DOCX)
